# Lipidopathy disrupts peripheral and central amyloid clearance in Alzheimer's disease: Where are our knowledge

**DOI:** 10.1016/j.ibneur.2025.01.004

**Published:** 2025-01-09

**Authors:** Shahram Darabi, Enam Alhagh Charkhat Gorgich, Fatemeh Moradi, Auob Rustamzadeh

**Affiliations:** a Cellular and Molecular Research Center, Research Institute for Prevention of Non-communicable Diseases, Department of Anatomical Sciences, School of Medicine, Qazvin University of Medical Sciences, Qazvin, Iran; b Department of Anatomy, School of Medicine, Iranshahr University of Medical Sciences, Iranshahr, Iran; c Department of Anatomy, School of Medicine, Iran University of Medical Sciences, Tehran, Iran

**Keywords:** Alzheimer's disease, Lipidopathy, Blood lipids, Amyloid-beta, Neurotoxicity

## Abstract

Amyloid-beta (Aβ) production is a normal physiological process, essential for neuronal function. However, an imbalance in Aβ production and clearance is the central pathological feature of Alzheimer’s disease (AD), leading to the accumulation of Aβ plaques in the brain. Low-density lipoprotein receptor-related protein 1 (LRP1) plays a critical role in both the central clearance of Aβ from the brain and its peripheral transport to visceral organs. Disruptions in these processes contribute to the accumulation of Aβ in the central nervous system (CNS) and the progression of AD. Recent research emphasizes the need for a broader focus on the systemic effects of organs outside the brain, particularly in the context of AD prevention and treatment. The contribution of peripheral systems, such as the liver, in Aβ clearance, is vital, given that Aβ levels in the plasma correlate closely with those in the brain. Consequently, targeting systemic processes, rather than focusing solely on the CNS, may offer promising therapeutic approaches. Furthermore, high-density lipoprotein (HDL) facilitates the formation of lipoprotein-amyloid complexes, which are important for Aβ transport and clearance, using proteins such as apolipoproteins E and J (ApoE and ApoJ) to form complexes that help manage Aβ accumulation. On the other hand, low-density lipoprotein (LDL) facilitates Aβ efflux from the brain by binding to LRP1, promoting its clearance. Given the relationship between lipid profiles and Aβ levels, along with lipid-modifying drugs, may be effective in managing Aβ accumulation and mitigating AD progression.

## Introduction

### Epidemiology, pathophysiology and current treatments of Alzheimer’s disease

Nearly 50 million people worldwide live with dementia and it is expected to reach about 152 million by 2050, putting tremendous socioeconomic pressure on patients, families and communities (Selkoe and Hardy, 2016, Wu et al., 2017). Studies show that people who only have amyloid-beta (Aβ) deposits, have an increasing prevalence of Alzheimer’s disease (AD), estimated to increase from 22.1 million in 2017–31.9 million in 2060 (Tahami Monfared et al., 2022). The incidence of AD in China is increasing and causing major medical and social problems. There are 15.07 million people aged 60 and over, with dementia, including 9.83 million with AD, 3.92 million with vascular dementia, and 1.32 million with other forms of dementia. The annual treatment cost of AD patients in China in 2015 was $167.74 billion, which is expected to reach $1.8 trillion, by 2050 (Ren et al., 2022). AD is currently the third leading cause of death, after cardiovascular disease and cancer (Ma et al., 2019). AD pathology is usually characterized by extracellular accumulations of Aβ peptide in Aβ plaques, and intracellular deposits of hyperphosphorylated tau (hp-Tau) protein, that form neurofibrillary tangles (Ma et al., 2019). The most famous cause of AD pathogenesis is the Aβ cascade hypothesis (Calabrò et al., 2021, Jellinger et al., 2008, Ewers et al., 2010), according to which, the primary onset of AD is caused by aberrant processing of amyloid precursor protein (APP) or presenilin (PS1 or PS2), leading to the formation of insoluble Aβ (Hardy and Allsop, 1991, Liu et al., 2019). APP, if cleavaged by alpha-secretase, produces soluble Aβ oligomers that have neuroprotective, neurogenic and synaptogenic functions (Mockett et al., 2017). However, when APP is cleavage by beta-secretase (BACE1), it produces insoluble Aβ_1–42_ fibrils that form Aβ plaques, which have pro-apoptotic and neurodegenerative roles and cause cognitive impairments (Saura et al., 2011, Moreno-Grau et al., 2015). The pathophysiology of AD is specifically related to the death of cholinergic neurons, located in the hippocampus and cortex of the brain that are mainly involved in memory and learning (H Ferreira-Vieira et al., 2016). To this date, existing drugs only relieve symptoms in AD patients, and cannot prevent disease progression. Among them, only four drugs named as donepezil, rivastigmine, galantamine, memantine and a combination drug called Namzaric, have been approved by the Food and Drug Administration (FDA) (Ma et al., 2019, Yiannopoulou and Papageorgiou, 2020). These drugs mainly relieve symptoms by maintaining acetylcholine levels and increasing neural connections, but have low efficacy in reversing the destructive effects of the disease and do not lead to definitive treatment. Currently, FDA-approved drugs for AD patients are mostly cholinesterase inhibitors (such as: donepezil, rivastigmine, and galantamine), and N-methyl-D-aspartate (NMDA) antagonists (such as memantine) (Yiannopoulou and Papageorgiou, 2020, Cummings et al., 2022). Most experts focus on the brain itself, for prevention and treatment of AD and pay less attention to the systemic effects of other organs on the course of AD progression. Research shows that the liver is the origin of Aβ deposits in the brain and plays a role in the peripheral clearance of Aβ in the blood circulation. New drugs synthesized for AD, should focus on increasing the peripheral clearance of Aβ protein, or specific liver products (Sagare et al., 2011a, Bassendine et al., 2020). Aβ production is a natural physiological process. The steady level of Aβ in the brain is determined by the balance between Aβ production and clearance, as the imbalance in the production/clearance ratio of Aβ protein, is the basis for its increase in AD. The Aβ hypothesis believes that Aβ accumulation is the causative factor of AD, leading to synaptic damage, hp-Tau, inflammation, OS, apoptosis and eventually damage and death of nerve cells (Sanabria-Castro et al., 2017).

Sagare et al. (2011) reported that the liver plays a critical role in the regulation of amyloid-beta (Aβ) levels, both as a site of peripheral synthesis and clearance. Studies highlight that Aβ produced peripherally can significantly contribute to brain amyloid pools through transport mechanisms mediated by the blood-brain barrier (BBB). Specifically, receptors such as RAGE (receptor for advanced glycation end products) facilitate the influx of circulating Aβ into the brain, while low-density lipoprotein receptor-related protein-1 (LRP1) mediates the efflux of Aβ from the brain to the circulation (Sagare et al., 2011a). Once in circulation, soluble LRP1 binds Aβ and directs it to the liver, where it undergoes systemic clearance. Research indicates that elevated levels of RAGE and reduced efficiency of LRP1 during aging or in AD models accelerate brain amyloid accumulation. This process links peripheral Aβ dynamics to cerebral deposition, emphasizing the interconnectedness of liver function and BBB mechanisms in maintaining Aβ balance. Moreover, peripheral reductions in Aβ have been shown to correlate with decreases in brain Aβ, suggesting that interventions targeting the liver or peripheral Aβ could offer therapeutic potential. However, further investigation is necessary to determine the efficacy of targeting hepatic Aβ production or transport mechanisms like RAGE in human Alzheimer’s disease. These insights underscore the liver's dual role in Aβ metabolism—both as a source and a site of clearance—and its critical interplay with the BBB in regulating brain amyloid levels (Bassendine et al., 2020, Sagare et al., 2007, Cheng et al., 2023). Lam et al. (2021) examines the role of the liver in producing and influencing brain amyloid-beta (Aβ) levels, providing evidence for a liver-to-brain pathway in AD. The study utilized a mouse model engineered to synthesize human Aβ specifically in the liver. These mice exhibited Alzheimer-like neurodegenerative phenotypes, including neurovascular inflammation, capillary dysfunction, and BBB disruption. This finding supports the hypothesis that peripheral Aβ, originating from the liver, can influence brain pathology through systemic circulation and vascular interactions (Lam et al., 2021). The research also highlights the association of Aβ with triglyceride-rich lipoproteins (TRLs) derived from the liver. Over 90 % of circulating Aβ is bound to these lipoproteins, which play a crucial role in its transport. TRL-Aβ has been implicated in the disruption of capillary integrity and the promotion of neurovascular inflammation, indicating a lipoprotein-mediated pathway that may increase AD pathology (Bassendine et al., 2020, Lam et al., 2021, Chandrashekar et al., 2024).

A key feature of late-onset Alzheimer's disease (LOAD) is a general disruption in Aβ clearance, which extends beyond neurons and microglia (Mawuenyega et al., 2010, Zolezzi et al., 2014). Peripheral clearance of Aβ from the brain occurs physiologically, accounting for 50 % of total Aβ clearance. This highlights the importance of peripheral Aβ clearance as a crucial mechanism to prevent its accumulation in the brain (Roberts et al., 2014). Within three minutes of Aβ_1–40_ and Aβ_1–42_ injection, the liver, the primary organ for plasma clearance, absorbs over 60 % of the peptides. The liver’s capacity to uptake, metabolize, and excrete Aβ significantly exceeds its physiological baseline, effectively clearing femtomolar Aβ levels in plasma and mitigating changes associated with aging and disease progression (Ghiso et al., 2004). Aβ is cleared from the brain parenchyma through multiple pathways, including lymphatic drainage via meningeal lymphatic vessels (glymphatic system) (Goodman et al., 2018), perineural (paradural) and perivascular spaces (Barisano et al., 2022, Gouveia-Freitas and Bastos-Leite, 2021, Wen et al., 2024), and transport across the BBB (by LRP1 and low-density lipoprotein receptor (LDLR)) (Shibata et al., 2000, Storck et al., 2016). In the bloodstream, LRP1 is the primary protein responsible for peripheral Aβ transport (Sagare et al., 2012a). Increased Aβ binding to LRP1 enhances its clearance, effectively preventing Aβ from re-entering the brain (Sagare et al., 2013). Amyloid-beta (Aβ) from the brain is cleared through arterial blood as it passes peripheral tissues like the liver, where LRP1 serves as the primary receptor for Aβ uptake (Xiang et al., 2015, Tamaki et al., 2006). LRP1 is involved in three stages of homeostatic control of Aβ clearance, including 1) cell surface LRP1 at the BBB and brain vascular cells, that clear Aβ by transferring it from the brain to the blood; 2) circulating LRP1 is a key peripheral endogen for Aβ that prevents brain exposure to Aβ (Sagare et al., 2007) and 3) LRP1 in the liver plays a role in systemic Aβ clearance (Zlokovic et al., 2010). LRP1 is expressed highly as an endocytic receptor in the liver, neurons, vascular smooth muscle cells in the CNS vessels, and glial cells (Kanekiyo et al., 2012).

### Peripheral lipidopathy as a potential risk factor for Alzheimer’s onset

Lipids, particularly cholesterol and its derivatives, play a key role in the pathophysiology of AD. The brain’s high cholesterol content is crucial for synaptic plasticity, emphasizing the importance of lipids in maintaining neural function and health (Pfrieger, 2003). Elevated brain cholesterol and its derivatives contribute to neuronal apoptosis, oxidative stress, and hp-Tau (2003, Panchal et al., 2010). The lipid profile of the cell membrane affects enzymes involved in APP processing and Aβ production. Dyslipidemia and high-fat diets (HFD) increase neuroinflammation, neurodegeneration, oxidative stress, hp-Tau, and Aβ accumulation, all key hallmarks of AD pathology (Wanamaker et al., 2015). Aβ disrupts cellular cholesterol distribution and esterification, impairing cholesterol homeostasis. It also increases hippocampal lipid synthesis, cholesterol uptake, and the accumulation of cholesterol and ceramides, which contribute to apoptotic pathways (Koudinov and Koudinova, 2005, Michikawa et al., 2001, Koudinov and Koudinova, 2001). AD can be triggered and progressed by acute and chronic systemic inflammation, even in individuals without genetic predisposition. Chronic inflammation outside the CNS, such as in the liver, can induce AD-like symptoms in the absence of genetic factors (Kim et al., 2016). Also, diet composition, duration, and timing significantly influence AD progression. HFD rich in saturated fatty acids (SFA) increase tau phosphorylation, while combining SFA and sucrose accelerates these effects within days. Western diets (WD), high in fat and cholesterol, promote amyloid and tau accumulation in the brain and disrupt gut function and nutrient absorption, further contributing to neurodegeneration (Calvo-Ochoa et al., 2014, Wiȩckowska-Gacek et al., 2021). Kim et al. (2016) highlight that a HFD triggers acute and chronic liver inflammation in wild-type (WT) and APP transgenic (APP-Tg) mice. This inflammation leads to immune cell infiltration, elevated pro-inflammatory cytokines, and exacerbated AD-related pathology, including accelerated Aβ plaque formation, increased plaque size, and heightened neuroinflammation. Notably, APP-Tg mice on an HFD showed four times more microglial activation than those on a standard diet (SD). Switching from an HFD to an SD reversed inflammation, reducing liver pathology, CNS inflammation, microglial activation, and Aβ plaque levels, particularly in the hippocampus and midbrain. These findings suggest a strong link between nonalcoholic fatty liver disease (NAFLD) and AD progression. While these improvements were observed in younger mice, older mice fed SD did not show similar benefits, emphasizing that aging alone is insufficient to account for astrocyte degradation or neuronal loss. The study v the role of dietary lipids and liver health in AD pathology, demonstrating that diet-induced liver dysfunction accelerates neurodegeneration. It also highlights the potential for dietary interventions to mitigate AD-related symptoms, particularly in the context of metabolic and inflammatory conditions like NAFLD (Kim et al., 2016). Chronic NAFLD in WT and APP-Tg mice on a HFD significantly reduced LRP1 expression in the CNS. This reduction was associated with worsened AD features, including neuronal and astrocyte loss, vascular damage, and fewer Aβ plaques, suggesting LRP1’s protective role in AD. The loss of glial cells and neurons, which are major sources of LRP1, likely contributes to this reduction, linking chronic liver dysfunction to neurodegeneration (Kim et al., 2016). ABCA1, a key transmembrane protein, utilizes ATP hydrolysis to facilitate the efflux of cholesterol and phospholipids from cell membranes. This function indirectly supports the clearance of amyloid-beta (Aβ) from the brain to the bloodstream, highlighting its critical role in maintaining lipid balance and contributing to AD pathology management (Darghal et al., 2010, Behl et al., 2021). ABCA1 facilitates Aβ clearance by enhancing the lipidation of Apolipoprotein E (ApoE), which binds Aβ more effectively when highly lipidated. This interaction improves Aβ transport efficiency, reducing its aggregation and promoting its removal at the neurovascular unit. By supporting Aβ efflux and preventing plaque formation, ABCA1 plays a protective role in maintaining brain health and mitigating AD pathology (Elali and Rivest, 2013). ABC transporters, particularly ABCA1 and ABCB1, play a critical role in clearing Aβ plaque from the brain. ABCA1, located at the BBB, facilitates Aβ transport via ApoE, enhancing its clearance by improving ApoE lipidation and reducing Aβ aggregation. These mechanisms reveal the importance of ABC transporters in maintaining brain health and mitigating AD pathology (Loeffler, 2024). ABCA1 deficiency is linked to increased amyloid-beta (Aβ) aggregation, contributing to AD pathology. ABCB1 (P-glycoprotein) also plays a key role in Aβ clearance, exporting Aβ from the brain into the bloodstream. In AD, reduced ABCB1 expression correlates with higher cerebral Aβ levels, suggesting its importance in preventing Aβ accumulation. Other ABC transporters, such as ABCA7, ABCC1, and ABCG1, also regulate Aβ levels and lipid metabolism, with ABCA7 specifically influencing phospholipid transport to high-density lipoprotein (HDL). These transporters are crucial for maintaining Aβ homeostasis in the brain (Chai et al., 2020a, Chai et al., 2020b). RAGE (Receptor for Advanced Glycation End-products) plays a key role in facilitating the entry of Aβ into the brain. As a multi-ligand receptor, RAGE interacts with various forms of Aβ, including monomers, oligomers, and fibrils. It has been shown in vitro to promote Aβ endocytosis and transcytosis across the BBB, contributing to Aβ accumulation in the central nervous system (CNS) (Zhou et al., 2021, Alkhalifa et al., 2023). RAGE is involved in regulating the cleavage APP through the GSK3β and p38 MAP kinase signaling pathways. This process contributes to Aβ production, linking RAGE to the progression of AD pathology (Tsoy et al., 2024). Increased RAGE expression promotes the influx of Aβ from the blood into the brain, exacerbating neurovascular dysfunction and neurodegeneration in AD. Inhibiting RAGE or blocking its interaction with Aβ has been shown to reduce Aβ accumulation in the brain, offering a potential therapeutic approach. Animal studies have demonstrated that RAGE inhibitors can slow Aβ pathology and cognitive decline (Tsoy et al., 2024, Rustamzadeh et al., 2024).

In AD, the progression involves Aβ accumulation, neuroinflammation, tau deposition, brain atrophy, and cognitive decline, eventually leading to dementia. Therefore, new treatments should target at least one of these key events. Additionally, disturbances in brain cholesterol homeostasis are implicated in several neurodegenerative diseases, including AD, suggesting that restoring cholesterol balance could be a promising therapeutic approach (Koudinov and Koudinova, 2005). Low HDL cholesterol levels are linked to a higher risk of dementia, while higher HDL levels are associated with larger hippocampal volume, which may prevent the onset of dementia (Wolf et al., 2004). HDL reduces Aβ toxicity by decreasing Aβ deposits and is crucial for synaptic maturation and the stability of synaptic plasticity (Pfrieger, 2003, Olesen and Dagø, 2000). Hypercholesterolemia significantly worsens AD pathology in animal models by impairing the processing of APP through alpha-secretase in both the hippocampus and frontal cortex (Shie et al., 2002). Low cholesterol levels reduce the volume of lipid rafts, where beta-secretase is predominantly located, thereby minimizing its involvement in APP processing (Ehehalt et al., 2003). Conversely, increasing cholesterol levels slightly decreases alpha-secretase activity while significantly enhancing beta-secretase activity, leading to increased Aβ production, elevated Aβ deposits, and subsequent neuronal toxicity (Wolozin, 2002). Memory impairment induced by high cholesterol is linked to dendritic damage, cholinergic dysfunction, inflammation, and increased Aβ and hp-Tau in the brain cortex, all of which are key diagnostic markers of AD (Granholm et al., 2008, Ullrich et al., 2010). Unlike cholesterol, oxidized cholesterol metabolites, or oxysterols, can cross the BBB in both directions. The main oxysterols in circulation are 24-hydroxycholesterol (24-HC) and 27-hydroxycholesterol (27-HC) (Loera-Valencia et al., 2019). Hypercholesterolemia increases 27-HC levels in the brain and blood, disrupting cholesterol metabolism and regulation. This contributes to accelerated AD progression, as plasma levels of 24-HC and 27-HC are notably higher in AD patients compared to healthy individuals (Choroszyński et al., 2022). Increased plasma levels of 24-HC predict cognitive impairment progression. Studies show that in individuals with cognitive decline, 24-HC and 27-HC levels correlate with Aβ_1–42_, and a decrease in 24-HC may signal neuronal death and brain atrophy (Dias et al., 2019). Evidence indicates that oxidative stress, inflammation, and disrupted cholesterol metabolism are key factors contributing to the progression of AD (Gamba et al., 2015). Under stress, cholesterol is converted to (oxidized) oxysterols and since the level of oxidative stress factors is high in AD patients, oxidized cholesterol is a driving force for AD. 27-HC is significantly absorbed from blood circulation to the brain (Gamba et al., 2019), and its level in the cortex of AD patients is higher than healthy individuals (Heverin et al., 2004).Therefore it may be the missing link between cholesterol disorders, and AD (Merino-Serrais et al., 2019). In contrast, the level of 24-HC, the main product of brain cholesterol metabolism, increases in the plasma of AD patients. This sterol is produced from cholesterol in the brain, by the activity of cholesterol-24-hydroxylase (CYP46) (a neural oxidative enzyme) (Papassotiropoulos et al., 2000).

**Fig. 1:**
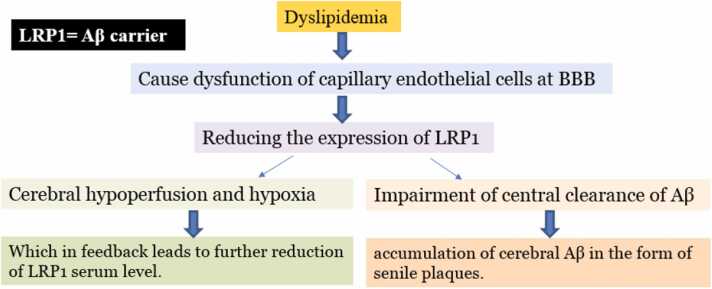
LRP1 mediates the uptake rate of Aβ in vascular smooth cells in the blood-brain barrier and may play a role in clearing amyloid peptides and modulating the disrupted balance of "the sink effect".

High levels of 24-HC reflect neurodegeneration (Kösch et al., 2001). Since lipids play an important role in neural function and synaptic plasticity (Pfrieger, 2003), abnormal increase in the level of cholesterol and its derivatives in the brain, causes induction of neuronal apoptosis, oxidative stress, hp-Tau, and also disrupts the activity of enzymes involved in APP processing and Aβ clearance (Ma et al., 2010). Dyslipidemia leads to BBB breakdown and disruption of brain cholesterol homeostasis, which can significantly increase Aβ plaques in the brain of AD patients (Panchal et al., 2010). The association between high levels of 27-HC and memory impairment has been shown in AD, as high levels of it cause disruption in neural structure and texture and consequently, cortical circuits. It has also been suggested that 27-HC is the main factor for memory impairment caused by increased peripheral cholesterol (Jahn et al., 2021) (Fig. 1). Liu et al. (2018) showed that Aβ_1–40_ and Aβ_1–42_ levels in the brain, significantly increase after administration of a diet containing 1.6 % cholesterol. Therefore, hyperlipidemia leads to increased levels of oxysterols, especially 27-HC, in peripheral blood and brain and also increased Aβ accumulation in the brain (Liu et al., 2018). In addition, studies show that adding 27-HC to cells in vitro, leads to increased Aβ production and hp-Tau (Loera-Valencia et al., 2019). Increased levels of 27-HC both in brain and blood circulation, affect brain cholesterol metabolism, which may also be important in AD progression (Merino-Serrais et al., 2019). Also, about 80 % of 24-HC present in the body, is found in the brain and must be transferred from CNS to plasma pool in order to be excreted by the liver (Vega et al., 2003). 24-HC may increase the activity of beta- or gamma-secretase enzymes that leads to Aβ production from APP and reducing APP processing through the non-amyloidogenic alpha-secretase pathway. After producing Aβ oligomers, cholesterol level can affect their aggregation status, in a way that high cholesterol level can make the binding of Aβ to the cell surface more difficult, and result in its accumulation and storage in the extracellular space (Wingo et al., 2019).

Bowman et al.’s (2012) study showed that dyslipidemia leads to brain inflammation and changes in the BBB integrity and that controlling TG and HDL can play an important role in changes in Alzheimer’s disease, from mild to moderate stage (Bowman et al., 2012). Martins et al. reported that AD and atherosclerosis patients have similar lipoprotein and cholesterol profiles (Martins et al., 2009). Significantly high levels of TC and LDL and low levels of HDL and ApoA to *Apo*I are significantly associated with AD progression (Merched et al., 2000). Yamchuen et al. (2014) showed that oxidized LDL, hyperlipidemia, and oxidative stress, are the key factors in AD onset, as 10–200 micrograms per milliliter of oxidized LDL in vitro, causes increased AChE activity and increased rate of intracellular ROS formation. They reported that oxidized LDL is cytotoxic and reduces cholinergic transmission rate in AD and that using anti-oxidant/anti-hyperlipidemic treatments to inhibit AChE, can be a suitable solution (Yamchuen et al., 2014). HDL prevents LDL oxidation and regulates cholesterol circulation, reduces atherosclerotic load in brain blood vessels, and reduces AD risk (Liu et al., 2020). Nunes et al. (2014) showed that plasma level of 27-HC is increased in individuals with low HDL, compared to the ones with high HDL (Nunes et al., 2014).

In addition, cholesterol flux was reduced in individuals with low HDL, compared to the group with high HDL, indicating an alternative pathway (especially participation in the Aβ deposit complex (Michikawa et al., 2001)) to maintain appropriate cellular cholesterol levels (Nunes et al., 2014). Moreover, Wolf et al.’s (2004) study showed that high serum levels of HDL, are associated with larger hippocampal volume and its low serum levels increase the risk of dementia, while LDL serum level and total cholesterol level showed no association (Wolf et al., 2004). Also, Kinno et al. (2019) showed that high HDL serum levels are not only associated with preserving memory function but also with increasing the thickness of the insular and opercular frontal cortex. Therefore, serum lipid levels of patients should be carefully evaluated in the clinic, as serum levels of HDL could be a biomarker for assessing memory function and structural features of the brain cortex (Kinno et al., 2019). Cholesterol balance is maintained by HDL function, as its role is to remove excess cholesterol by transferring it to the liver. An increase in HDL leads to increased synthesis of ApoA to *Apo*I. In the cleavage pathway of APP, apolipoproteins play a role. In particular, ApoE controls γ-secretase cleavage and lowers Aβ40 levels, which ultimately reduces the formation of senile plaque. Since the apoA-I and HDL mediate the process of cholesterol exit, they lead to increased membrane fluidity which increases non-amyloidogenic processing by alpha-secretase to produce soluble Aβ, which is cleared by the liver, and otherwise, Aβ plaques are formed (Koldamova et al., 2001). Also, apoA-I can bind APP to the cell surface and prevent cytosolic processing of APP, which is necessary for the activity of β- and γ-secretase, to reduce the production of neurotoxic insoluble Aβ (Ciccone et al., 2020). Another key role of HDL is increasing Aβ clearance, as laboratory studies show that apoA-I interacts with Aβ and prevents its aggregation (Koldamova et al., 2001). In addition, the apoA-I - HDL complex has a high tendency to bind to Aβ and clear it through BBB (Sagare et al., 2012b). Slot et al.’s (2017) study showed that high cerebrospinal fluid (CSF) and low plasma levels of apoA-I, are associated with increased risk of disease progression in individuals with MCI and AD (Slot et al., 2017). A phase II clinical trial, in which AD patients were treated with Apabetalone (RVX-208) (a molecule that stimulates APOA-I gene expression) for 12 weeks, showed that plasma levels of Aβ increased compared to the baseline levels (McNeill, 2010). Increased plasma levels of apoA-I are associated with increased clearance of Aβ from peripheral circulation (Mancini et al., 2016). The apoA-I - HDL complex can also increase Aβ clearance through the brain vessels. Robert et al. (2017) demonstrated that HDL crosses the BBB to enhance Aβ clearance, reducing brain Aβ load through the peripheral "sink effect." Elevated HDL levels may alleviate AD symptoms by reducing Aβ production, preventing aggregation, and accelerating clearance via receptors such as LRP-1 in glial cells and the BBB. These findings support HDL-focused strategies as potential AD therapies (Robert et al., 2017, Stukas et al., 2014, Jin et al., 2021). Due to its unique structures such as a hydrophobic lipid core and a hydrophilic apolipoprotein shell, HDL can act as an ideal mediator for transporting hydrophobic drugs, gene drugs, peptides, or imaging agents, through various combinations such as hydrophobic core loading, surface loading, and covalent modification (Gupta et al., 2021). Cross-sectional studies show that serum levels of HDL and carrier HDL (in plasma), are significantly lower in AD patients and are inversely correlated with MMSE scores (Shih et al., 2014).

**Fig. 2:**
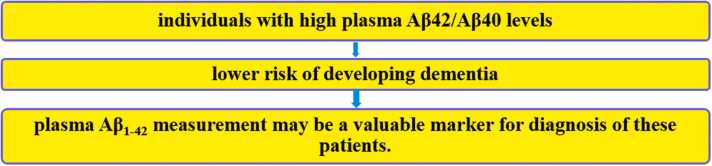
The low concentration of Aβ_1–42_ in blood circulation indicates the increase of Aβ in the brain, the formation of amyloid plaques, and cognitive deficits in Alzheimer disease.

There is a direct association between high levels of TG, LDL, and TC, and low levels of Aβ_1–42_ in the plasma of AD patients (Iqbal et al., 2020), while high levels of HDL are associated with increased plasma levels of Aβ_1–42_ (Bernath et al., 2020). Also, Choi et al.’s (2016) study showed that high serum levels of TG are associated with high brain Aβ deposition (Choi et al., 2016). Animal studies showed that serum level of TG is high before Aβ deposition in the brain (Burgess et al., 2006). The lack of peroxisome proliferator-activated receptor gamma (PPARγ) receptor, is the probable reason for increasing TG which leads to Aβ accumulation and cognitive impairment (Kershaw et al., 2007). This receptor regulates adipocyte triacylglycerol lipase (ATL) activity, and facilitates Aβ clearance from the brain (Mandrekar-Colucci et al., 2012) (Fig. 2).

### Aβ clearance mechanisms

Numerous studies show that low concentration of Aβ_1–42_ in blood circulation (serum and plasma), indicates increased Aβ in the brain, formation of Aβ deposits, and cognitive impairment, in AD patients (Risacher et al., 2019, Pérez-Grijalba et al., 2019, Cianflone et al., 2021, Yamamoto et al., 2014). Lambert et al.’s (2009) study, which was conducted prospectively in France, showed that individuals who have higher plasma Aβ_1–42_/Aβ_1–40_ levels, have a lower risk of dementia and concluded that plasma Aβ concentration may be a key indicator of the risk of dementia (Lambert et al., 2009). Although the exact mechanism of this correlation in AD is not well understood, reports show that LRP1 regulates brain (central) and systemic (peripheral) Aβ clearance, in AD (Zlokovic et al., 2010). Risacher et al.’s (2019) study suggested that measuring plasma Aβ can be a useful biomarker for predicting brain Aβ status (Risacher et al., 2019). de Wolf etal. (2020) analyzed plasma samples and clinical data from 4444 dementia-free participants in Rotterdam between 2002 and 2016, measuring tau, neurofilament light chain (NFL), and Aβ1–42 biomarkers. Over 14 years, 549 participants developed dementia, including 374 with AD. Higher plasma Aβ_1–42_ levels correlated with a lower AD incidence, while elevated NFL levels were associated with AD development. Notably, Aβ_1–42_ levels began declining years before an AD diagnosis, emphasizing its potential as an early biomarker for monitoring disease progression (de Wolf et al., 2020). Wei et al.’s (2022) study showed that administration of simvastatin 40 mg per day in hyperlipidemic patients for 12 weeks, can significantly increase plasma levels of Aβ_1–42_ compared to placebo. They reported that increased plasma Aβ_1–42_ is directly related to decreased triacylglycerol levels and RAGE (a peptide that has an opposite role to LRP1 and causes transfer of Aβ from blood circulation to the brain) (Wei et al., 2022). Sagare et al. (2007) reported that the plasma level of LRP1 is decreased in AD patients, compared to healthy individuals. Also, in individuals with normal cognition, 70–90 percent of Aβ is bound to plasma LRP1, but in AD patients LRP1 peptide is oxidized and has less binding tendency to Aβ (Sagare et al., 2007). Another study in 2013 showed that improving Aβ binding to plasma LRP1 increases Aβ clearance efficiency (Sagare et al., 2013) and reducing peripheral Aβ level causes discharge of brain Aβ, through BBB (Zlokovic, 2011). Sehgal et al. (2012) showed that a “Withania somnifera” present in grapes, plays an important role in clearing Aβ in Alzheimer’s mouse model through upregulation of LRP1 and improving Aβ binding to LRP1 (Sehgal et al., 2012).

### Peripheral clearance of Aβ

Mediators and transporters are essential for clearing Aβ, a protein central to AD pathology. They facilitate Aβ removal from the brain, maintaining homeostasis and preventing its buildup. Soluble LRP1 is a key transporter in peripheral Aβ clearance (Lai et al., 2021). LRP1 is expressed in various tissues, including the liver, and is responsible for binding and internalizing Aβ for subsequent degradation. ATP-binding cassette (ABC) transporters, such as ABCA1 and ABCG1, are also involved in Aβ peripheral clearance. These transporters promote the efflux of Aβ from the brain to the periphery, where it can be cleared by the liver and kidneys (Behl et al., 2021). In addition, P-glycoprotein (P-gp) also plays a role in Aβ peripheral clearance. P-gp is an efflux transporter expressed in the blood-brain barrier, and it actively transports Aβ out of the brain into the bloodstream (Arduino et al., 2020). Dysfunction of mediators like LRP1 impairs Aβ clearance, leading to brain accumulation and contributing to AD. Reduced LRP1 expression in patients correlates with increased Aβ deposition. Understanding these mechanisms is critical for developing therapies to enhance Aβ clearance. Future strategies, such as targeting LRP1 upregulation or novel transporter modulators, hold promise for advancing personalized treatments and mitigating disease progression (Zhou et al., 2021).

### Central clearance of Aβ

One important mediator involved in Aβ central clearance is the insulin-degrading enzyme (IDE). IDE is responsible for degrading Aβ within the brain, preventing its accumulation and subsequent formation of plaques (Fernández-de Frutos et al., 2019). Another mediator is neprilysin, also known as neutral endopeptidase (NEP). NEP is an enzyme that degrades Aβ and is expressed in various brain regions. It plays a crucial role in the clearance of Aβ and its dysfunction has been implicated in Alzheimer's disease pathology (Yamamoto et al., 2022). Astrocytes, a type of glial cell in the brain, express several transporters involved in Aβ central clearance. For example, the ATP-binding cassette transporter ABCB1 is expressed on the surface of astrocytes and actively transports Aβ (Darghal et al., 2010). RAGE expressed on microglia and astrocytes facilitates the internalization and degradation of Aβ within the brain (Gospodarska et al., 2011). Studies have shown that decreased expression or activity of IDE and NEP, as well as alterations in astrocytic transporters, are associated with increased Aβ levels and plaque formation in Alzheimer's disease (Lu et al., 2020).Table 1

**Table 1:** Effects of peripheral dyslipidemia on Aβ clearance.

**Author**	**Patients properties**	**Lipid profile**	**changes of Aβ level**	**Main findings**
Hu et al. (2020) (Hu et al., 2020)	Participants with high and normal blood pressure	Higher TC and LDL-c Lower LDL-c	Elevation of plasma Aβ1–42 level normal blood pressure Elevation of plasma Aβ1–40 in high blood pressure.	Levels of blood lipids and plasma Aβ were confounded by blood pressure.
Prasanthi et al. (2008) (Prasanthi et al., 2008)	White male rabbits that received a cholesterol-enriched diet.	Increase in Aβ levels and BACE1 as well as RAGE and decrease in levels of LRP−1 in the cortex and hippocampus	Hypercholesterolemia increases Aβ production, an effect that is associated with increased levels of BACE1 and RAGE and reduced levels of IDE and LRP−1.	Hypercholesterolemia: induces Aβ accumulation via upregulation of the BACE1 enzyme and increase in levels of RAGE. Diminish of Aβ clearance by a decrease in levels of LRP−1.
She et al. (2021) She et al., (2021)	Participants with high and normal blood pressure	-	Increased plasma Aβ1–40 levels in high blood pressure	Elevated BP levels were associated with increased plasma Aβ1–40 levels in middle-aged and elderly ApoE ε4 non-carriers
			Aβ1–42 level had no significant difference between the high BP group and normal BP group	

### Production and degradation of Aβ, and factors affecting it in the brain and periphery

In AD, amyloid deposition begins in the neocortex during early stages. It then spreads to the entorhinal cortex, hippocampal CA1 region, cingulate gyrus, and amygdala. In advanced stages, Aβ plaques extend to the striatum, thalamus, hypothalamus, and brainstem regions (Koychev et al., 2020). It seems that Aβ neuronal toxicity occurs through initiating local inflammatory responses and apoptotic pathways, and is caused by oxidative effects of Aβ (Agostinho et al., 2010). Continuous removal of Aβ from the brain is very important to prevent the formation of Aβ plaques, and AD progression (Tarasoff-Conway et al., 2015). Soluble Aβ peptides can be absorbed by microglia and astrocyte phagocytosis. Another way is through clearance of bulk flow of interstitial fluid (ISF), by CSF drainage and perivascular clearance. However, it seems that most of Aβ peptides are removed through active transport across BBB, as the main clearance pathway (McIntee et al., 2016).

The main receptors for the uptake and transport of Aβ fragments across the BBB are the RAGE receptor (the main receptor responsible for transporting Aβ from blood to brain parenchyma, across the BBB), and LRP1 (the main receptor responsible for transporting Aβ from brain parenchyma to blood, across the BBB) (Sagare et al., 2012a). The precursor protein of LRP1 with a weight of 600 kilodaltons, is synthesized in the endoplasmic reticulum and cleaved by the Golgi apparatus, which is also called CD91 or alpha-2 macroglobulin receptor (Herz and Strickland, 2001). The gene encoding LRP1 is located on the long arm of chromosome 12 (12q13-q14) (Herz et al., 1988). LRP1 is expressed in many types of cells, including brain endothelium, neurons, smooth muscle cells, astrocytes, macrophages, fibroblasts, and liver cells (Moestrup et al., 1992). Studies have shown that 50 % of Aβ is transferred from the brain to blood by transcytosis across the BBB, mediated by LRP1. Also, Aβ degradation occurs in smooth muscle cells of vessels (Tanzi et al., 2004). Enzymatic degradation can be extracellular or intracellular. Extracellular degradation of Aβ mainly depends on protein-degrading enzymes secreted from cells, including neprilysin, insulin-degrading enzymes, and endothelin-converting enzymes. Aβ can also be absorbed by neurons, microglia, and astrocytes. It could also be degraded by proteases and lysosomes (Tarasoff-Conway et al., 2015). The continuous and slow flow of ISF (which surrounds neurons) to CSF (which surrounds the brain), followed by drainage to blood in the perivascular space, constitutes 10–15 % of total Aβ clearance in mice (Parodi-Rullán et al., 2020). Approximately 70 % of Aβ in plasma, is directly bound to soluble LRP (sLRP) which forms a significant part of the “sink effect” endogenous circuit, which its main function is clearance of peripheral Aβ. sLRP reduces free Aβ levels in blood circulation and causes increased clearance mediated by LRP in the cell surface, which occurs from the BBB toward outside the brain (Sagare et al., 2007). Cholesterol determines the amount of the release of sLRP1. Several studies have shown that sLRP1 can cause removal of Aβ from the brain to blood, through transcytosis from BBB (Sagare et al., 2007, Sagare et al., 2013, Sagare et al., 2011b), which makes it a suitable biomarker for monitoring AD (Shinohara et al., 2017). Several drugs including statins, pioglitazone, winter cherry, and Chinese Linguizhugan tea, increase LRP1 expression in the brain or liver, causing increased clearance of Aβ (Sehgal et al., 2012, Hu et al., 2018, Seok et al., 2019). In AD patients, LRP1 expression decreases in brain vessels (Osgood et al., 2017) but increases in neurons and active astrocytes around senile plaques (Arélin et al., 2002). LRP1 is important for the rapid removal of Aβ from the brain, due to its ability for fast endocytosis, compared to other members of the LDLR receptor family (Li et al., 2001). Bu et al. (1994) showed that LRP1 is abundantly expressed in the cell body and proximal processes of cortical and hippocampal neurons (Bu et al., 1994). LRP1 binds to Aβ_1–42_ (directly or through apoE) and facilitates clearance of Aβ from the brain (Storck et al., 2016, Shinohara et al., 2017, Yamada et al., 2008, Kanekiyo and Bu, 2014). In AD patients and elderly people, brain LRP1 level decreases significantly, and has an inverse relationship with the age of AD onset, which indicates that reduced LRP1 function causes cognitive decline (Kang et al., 2000). Another study showed that reduced LRP1 expression significantly decreases the uptake of Aβ_1–42_ peptide in neurons. Impairment in LRP1 endocytic function causes increased accumulation of Aβ_1–42_ in neurons and ultimately leads to increased cellular toxicity (Fuentealba et al., 2010).

## Conclusion

Recent advances in blood-based biomarkers have improved the diagnosis of pre-symptomatic AD, enabling better drug design. Current research focuses on therapies targeting the early stages of AD, with an emphasis on multi-factor approaches (e.g., anti-Aβ, anti-tau, anti-inflammatory). Effective biomarkers must reflect preclinical disease manifestations, and findings from animal models should translate effectively to clinical settings. Elevated serum Aβ levels following increased brain Aβ clearance correlate with cognitive improvement, suggesting that maintaining optimal HDL levels may enhance antioxidant factors and support neuroprotection. HDL function measurement could serve as a promising prognostic marker for tailoring AD therapies (Hampel et al., 2018). Assessing HDL for monitoring Aβ modulation offers a promising approach for early AD diagnosis, as detecting Aβ levels before neuronal damage and tissue atrophy is critical for effective treatment. Dyslipidemia, particularly abnormal HDL and triglyceride changes, contributes to AD progression and treatment resistance, a condition known as peripheral Lipidopathy, which is a key factor in sporadic AD. The pathological mechanisms of AD involve Aβ processing, clearance, and transport across the BBB. Given the close relationship between lipid profiles and serum Aβ levels, lifestyle changes and lipid-modifying drugs may be beneficial. However, further research is needed to clarify HDL’s role in AD pathogenesis, focusing on two unresolved questions: 1) the causal relationship between HDL and AD, and 2) the protective mechanisms of HDL against AD. Although aducanumab received FDA approval in 2022 for its impact on Aβ plaque load, peripheral Aβ clearance must be balanced for lasting efficacy. Thus, drug design should target both peripheral clearance and the balance of Aβ production and clearance. Given the poor outcomes of Aβ-targeted therapies, multi-dimensional treatments addressing oxidative stress and neuroinflammation offer a promising strategy for AD therapy (Šerý et al., 2013). Since blood biomarkers provide in vivo and quantitative presentation of AD pathophysiology, they are now included as diagnostic components of AD in modern research, and recommended to be used in clinical trials (Hampel et al., 2018, Hampel et al., 2010). Current diagnostic methods for AD, such as lumbar puncture and PET imaging for Aβ and tau, allow direct observation of AD pathogenesis but are not suitable for routine screening due to their invasiveness, cost, and limited availability. Ideal diagnostic and therapeutic monitoring tools should be non-invasive, affordable, and accessible in public healthcare settings. Advances in omics technologies and the creation of dedicated databases will help address methodological challenges in future research. Despite cholesterol’s known impact on AD mediators like Aβ and tau, the cellular pathways linking cholesterol metabolism disorders to AD remain unclear. Key questions include how cholesterol metabolism is altered in specific brain cells, how lipid transfer and metabolism genes affect neurons and glial cells, and how these changes contribute to amyloidosis and tauopathy in AD. Technological advancements, including gene knock-out models and Aβ/tau-specific radioisotope tracers, could aid diagnosis and treatment. However, measuring cholesterol levels in later stages of AD may have limited clinical impact due to irreversible neural damage. Therefore, researchers should focus on utilizing validated biomarkers in serum, cerebrospinal fluid, and neuropsychological tests for future epidemiological studies.

## Ethics approval and consent to participate

Not applicable.

## Funding

The authors did not receive any funding.

## CRediT authorship contribution statement

**Auob Rustamzadeh:** Writing – review & editing, Writing – original draft, Visualization, Validation, Supervision, Resources, Project administration, Methodology, Investigation, Formal analysis, Data curation. **Shahram Darabi:** Writing – review & editing, Writing – original draft, Supervision, Software, Resources, Project administration, Methodology, Investigation, Conceptualization. **Enam Alhagh Charkhat Gorgich:** Writing – review & editing, Writing – original draft, Software, Methodology, Investigation, Formal analysis, Conceptualization. **Fatemeh Moradi:** Writing – review & editing, Writing – original draft, Visualization, Software, Resources, Methodology, Investigation, Formal analysis, Data curation.

## Declaration of Competing Interest

The Authors declare no competing financial or non-financial interests directly or indirectly related to the work submitted for publication.
